# Changes in aortic blood flow induced by passive leg raising predict fluid responsiveness in critically ill patients

**DOI:** 10.1186/cc5044

**Published:** 2006-09-13

**Authors:** A Lafanechère, F Pène, C Goulenok, A Delahaye, V Mallet, G Choukroun, JD Chiche, JP Mira, A Cariou

**Affiliations:** 1 Medical Intensive Care Unit, Cochin Hospital, APHP, Université Paris Descartes, 27, rue du Faubourg Saint Jacques, 75679 Paris Cedex 14, France

## Abstract

**Introduction:**

Esophageal Doppler provides a continuous and non-invasive estimate of descending aortic blood flow (ABF) and corrected left ventricular ejection time (LVETc). Considering passive leg raising (PLR) as a reversible volume expansion (VE), we compared the relative abilities of PLR-induced ABF variations, LVETc and respiratory pulsed pressure variations (ΔPP) to predict fluid responsiveness.

**Methods:**

We studied 22 critically ill patients in acute circulatory failure in the supine position, during PLR, back to the supine position and after two consecutive VEs of 250 ml of saline. Responders were defined by an increase in ABF induced by 500 ml VE of more than 15%.

**Results:**

Ten patients were responders and 12 were non-responders. In responders, the increase in ABF induced by PLR was similar to that induced by a 250 ml VE (16% versus 20%; *p *= 0.15). A PLR-induced increase in ABF of more than 8% predicted fluid responsiveness with a sensitivity of 90% and a specificity of 83%. Corresponding positive and negative predictive values (PPV and NPV, respectively) were 82% and 91%, respectively. A ΔPP threshold value of 12% predicted fluid responsiveness with a sensitivity of 70% and a specificity of 92%. Corresponding PPV and NPV were 87% and 78%, respectively. A LVETc of 245 ms or less predicted fluid responsiveness with a sensitivity of 70%, and a specificity of 67%. Corresponding PPV and NPV were 60% and 66%, respectively.

**Conclusion:**

The PLR-induced increase in ABF and a ΔPP of more than 12% offer similar predictive values in predicting fluid responsiveness. An isolated basal LVETc value is not a reliable criterion for predicting response to fluid loading.

## Introduction

In sedated, mechanically ventilated patients, fluid responsiveness can be efficiently predicted by assessing the respiratory changes in arterial pressure. However, in some patients it might be attractive to use a reversible maneuver that mimics a fluid challenge and to assess its hemodynamic consequence directly.

Esophageal Doppler (ED) provides a continuous measurement of the descending aortic blood flow (ABF), which constitutes a reliable indicator of global cardiac output [1]. This device also offers a measurement of the left ventricular ejection time corrected for heart rate (LVETc), a value that has been proposed as a criterion of static preload [2,3]. Moreover, ED may provide dynamic criteria to predict fluid responsiveness, such as the respiratory variation in peak aortic velocity or in ABF. These criteria are based on variations in stroke volume (SV) induced by cyclic respiratory changes in ventricular preload, in a similar manner to the variation in arterial pulse pressure (ΔPP) [4]. Monnet and colleagues [5] recently demonstrated that these respiratory changes in ABF provide a better prediction of fluid responsiveness than LVETc does. However, such measurements require sophisticated software to analyse computerized signals, and such software is not available for currently commercialized devices.

Passive leg raising (PLR) is a simple reversible maneuver that mimics a rapid fluid loading. It transiently and reversibly increases venous return by shifting venous blood from the legs to the intrathoracic compartment [6,7]. PLR increases right and left ventricular preload, which may lead to an increase in SV and cardiac output [8-10]. Boulain and colleagues found that PLR resulted in increased SV measured by thermodilution only in the subset of mechanically ventilated patients who subsequently increased their SV in response to volume expansion (VE) [11]. Accordingly, ΔPP during PLR was proposed as a non-invasive predictor of responsiveness to preload in patients receiving mechanical ventilation. However, it can be hypothesized that the predictive value of PLR could be improved through the use of a more direct estimate of SV than is possible by respiratory changes in pulse pressure. By providing real-time monitoring of ABF, the ED is an attractive method to monitor cardiac output changes [12]. Thus the effects of PLR on ABF could be a simple method to predict preload-responsiveness.

Therefore, the aim of our study was to evaluate and compare the relative ability of variations of ABF during PLR, LVETc and ΔPP to predict fluid responsiveness in mechanically-ventilated patients with acute circulatory failure requiring VE.

## Materials and methods

### Patients

The study patients were (1) intubated, mechanically ventilated in a volume-controlled mode, and fully sedated, (2) in acute circulatory failure, (3) receiving stable doses of vasopressive drugs, (4) monitored by ED and a radial or femoral arterial catheter and (5) requiring VE according to the attending physician.

Acute circulatory failure was defined as (1) a systolic blood pressure lesser than 90 mmHg (or a decrease of more than 50 mmHg in previously hypertensive patients) or the need for vasopressive drugs, (2) a urine output below 0.5 ml/kg/minute for at least two hours, (3) tachycardia (heart rate > 100 bpm) and (4) the presence of skin mottling.

Fluid loading requirements were based on the presence of at least one clinical sign of acute circulatory failure and/or associated signs of visceral hypoperfusion, including signs of renal dysfunction, hepatic dysfunction, and/or increased arterial blood lactate, in the absence of a contraindication for a fluid challenge. Contraindication for a fluid challenge was defined as a life-threatening hypoxemia and by the evidence of blood volume overload and/or of hydrostatic pulmonary oedema. Patients with spontaneous breathing activity, cardiac arrhythmias or patients having contraindication for the use of ED (that is to say, known or suspected oesophageal ulcer, mycosis, malformation, varicose or tumour) were excluded, as were patients with incapacity to practice PLR.

### Measurements

ED measurements were obtained by using the Hemosonic 100™ device (Arrow Intl, Everett, Ma., USA). This device enables continuous measurement of descending thoracic aorta blood velocity (Doppler transducer) and measures the real aortic diameter (M-mode echo transducer) [13]. ABF is continuously calculated from aortic blood velocity and diameter echo signals and its mean value is calculated and averaged over a 10-second period. LVETc is calculated by dividing systolic flow time by the square root of the cycle time.

Pulse pressure is the difference between systolic and diastolic arterial pressure. Maximum (PP_max_) and minimum (PP_min_) values were determined over a whole respiratory cycle. ΔPP was calculated as previously described [4]: ΔPP (%) = 100 × {(PP_max _ΔPP_min_)/([PP_max _+ PP_min_]/2)}. Three respiratory cycles were used and averaged to calculate ΔPP.

### Study protocol

**Figure 1:**
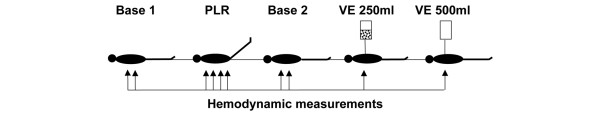
Study protocol.

The protocol sequence is shown in Figure 1. Heart rate, systolic arterial pressure, mean arterial pressure, diastolic arterial pressure, ABF and LVETc were recorded in the following consecutive steps:

1. Twice over two minutes at one-minute intervals, in the supine position (Base 1).

2. Four times over four minutes at one-minute intervals, during PLR (lower limbs were lifted in a straight manner by an assistant to a 45°).

3. Twice over two minutes at one-minute intervals, in the supine position (Base 2).

4. Once after a fluid loading of 250 ml of 0.9% sodium chloride (VE 250 ml).

5. Once after a second fluid loading of 250 ml of 0.9% sodium chloride (VE 500 ml), which was begun immediately after the first 250 ml of fluid loading.

The ED probe was repositioned if aortic blood velocity or aortic diameter signals were lost during the procedure. No treatments (such as ventilatory settings or vasopressive drugs dosage) that might alter hemodynamic status were given during the study period.

Because ED monitoring and VE are part of routine care in patients with acute circulatory failure treated in our unit, French law authorizes the conduct of this kind of observational study without informed consent. Whenever possible, each patient or next of kin was informed and consented to the use of registered data.

### Statistical analysis

After completion of the study protocol, patients were divided into two groups: responders and non-responders to fluid loading. A patient was classified as a responder when an increase of more than 15% in ABF was induced by fluid loading between Base 2 and VE 500 ml. Base 1, PLR and Base 2 sequences contain two or four consecutive hemodynamic measurements. An average of these multiple measurements was calculated for each sequence and used in the statistical analysis.

Given the small number of patients, results are expressed as median (interquartile range) or number and percentage. Non-parametric tests were used for comparisons. The Friedman test followed by the Wilcoxon rank sum test was performed to detect changes over time (before and after PLR and VE) within the same group (responder or non-responder). The comparisons of hemodynamic parameters between responders and non-responders were assessed with the Mann–Whitney *U *test. Linear correlations were tested with the Spearman rank method. Receiver operating characteristic (ROC) curves were generated for PLR-induced changes in ABF, LVETc and ΔPP by varying the discriminating threshold of each parameter. The area under each ROC curve (AUC) was calculated and expressed as AUC ± SD. *p *< 0.05 was chosen as being significant. Statistical analysis was performed with SPSS version 12.0 (SPSS Inc., Chicago, IL, USA).

## Results

### Study population

Twenty-three patients were screened for inclusion, and 22 were studied; their clinical characteristics are reported in Table 1. One patient had to be excluded because it was not possible to obtain an aortic diameter signal. The underlying diseases were as follows: hypertension (*n *= 6), ischemic cardiomyopathy (*n *= 8), dilated cardiomyopathy (*n *= 1), chronic obstructive pulmonary disease (*n *= 4), chronic renal failure (*n *= 1) and diabetes (*n *= 2).

### Effects of passive leg raising and volume expansion on aortic blood flow

Ten patients were responders (increase in ABF of more than 15% induced by VE between Base 2 and VE 500 ml), and 12 were non-responders. Hemodynamic data for responders and non-responders are presented in Tables 2 and 3, respectively.

Basal values of heart rate and arterial pressure were not different between the two groups. However, Base 1 ABF was lower in responders than in non-responders (2.4 (1.8 to 4.0) l/minute versus 4.2 (3.3 to 4.9) l/minute; *p *= 0.03) and increased significantly during PLR (Table 2). After VE 250 ml and after VE 500 ml, ABF increased significantly and heart rate decreased significantly in comparison with Base 2 (Table 2). In non-responders, heart rate, arterial pressure and ABF variations during PLR or VE were not statistically significant (Table 3).

### Ability of passive leg raising maneuver to predict fluid responsiveness

**Figure 2:**
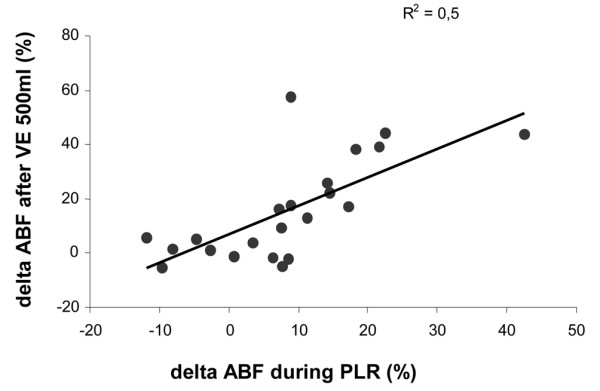
Relationship between changes in ABF induced by PLR and VE. Abbreviations: ABF = aortic blood flow; PLR = passive leg raising; VE = volume expansion. Results are expressed as percentage variation from Base 1 value for PLR and from Base 2 value for VE.

**Figure 3:**
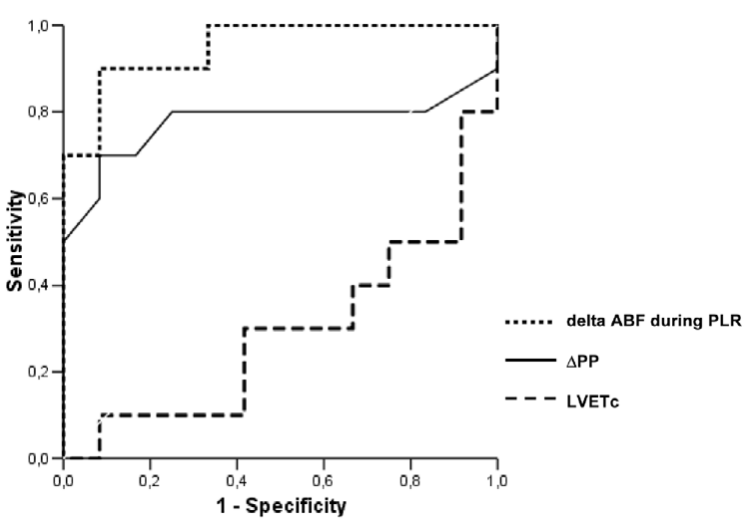
ROC curves comparing delta ABF, LVETc and ΔPP to discriminate responders and non-responders. Abbreviations: ROC = Receiver Operating Characteristic; ABF = aortic blood flow; PLR = passive leg raising; LVETc = left ventricular ejection time corrected for heart rate; ΔPP = respiratory variation of pulse pressure.

Considering all patients, the increase in ABF induced by PLR was correlated positively with that induced by VE 500 ml (*r*^2 ^= 0.5, *p *< 0.0001; Figure 2). In responders, the PLR-induced increase in ABF was similar to that induced by a 250 ml VE (16% (10 to 21%) versus 20% (1 to 30%), *p *= 0.15). The most discriminant value of changes in ABF that was able to predict the absence or presence of fluid responsiveness was assessed with the ROC curve (Figure 3). Thus, a PLR-induced increase in ABF greater than 8% predicted the response to subsequent VE with a sensitivity of 90% and a specificity of 83%. The corresponding positive and negative predictive values (PPV and NPV, respectively) were 82% and 91%, respectively.

### Ability of variation in arterial pulse pressure to predict fluid responsiveness

**Figure 4:**
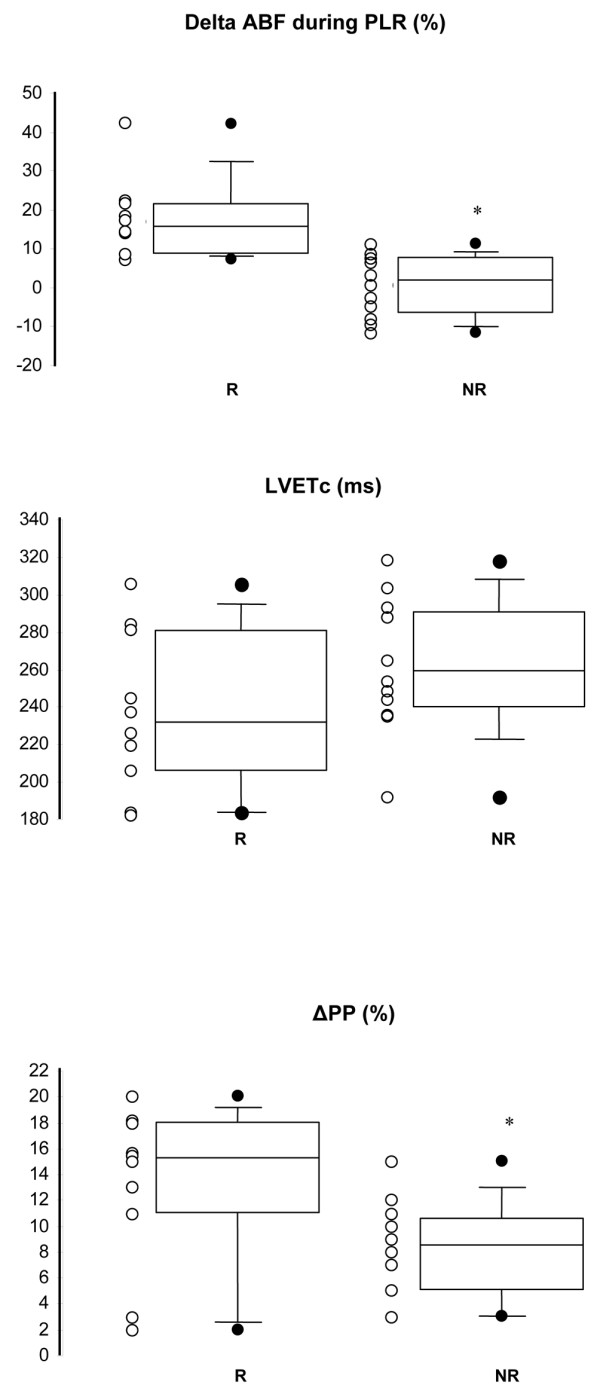
Boxplots and individual values of change in ABF, LVETc and ΔPP in responders and non-responders. Abbreviations: ABF = aortic blood flow; LVETc = left ventricular ejection time corrected for heart rate; ΔPP = respiratory variation of pulse pressure. Asterisk, *p *< 0.05 for responders versus non-responders.

In responders, basal ΔPP was greater than in non-responders (15% (12 to 17%) versus 9% (5 to 10%), *p *= 0.03; Figure 4). Its decrease during PLR and VE 500 ml was significant (Table 2). In non-responders, ΔPP did not change significantly after VE 500 ml. It decreased significantly during PLR; however, this change was not clinically relevant (9% (5 to 10%) versus 7% (5 to 7%), *p *= 0.024; Table 3). A basal ΔPP threshold value of 12% predicted fluid responsiveness with a sensitivity of 70% and a specificity of 92%. The corresponding PPV and NPV were 87%and 78%, respectively.

### Ability of left ventricular ejection time to predict fluid responsiveness

Base 1 LVETc was not statistically different between responders and non-responders (232 ms (209 to 272 ms) versus 259 ms (242 to 289 ms), *p *= 0.10; Figure 4). However, in responders, LVETc increased during PLR, VE 250 ml and VE 500 ml, whereas it did not change significantly in non-responders (Tables 2 and 3). An LVETc of 245 ms or less predicted fluid responsiveness with a sensitivity of 70% and a specificity of 67%. The corresponding VPP and VPN were 60% and 66%, respectively.

As shown in Figure 3, the AUC for PLR-induced changes in ABF and for ΔPP were 0.95 ± 0.04 and 0.78 ± 0.12, respectively, whereas the AUC for LVETc was 0.29 ± 0.12; these data suggest that LVETc does not accurately predict fluid responsiveness.

## Discussion

Our study demonstrates mainly that fluid responsiveness in mechanically ventilated patients with acute circulatory failure can be efficiently predicted by assessing the effects of PLR on ABF monitored by ED. We found that the PLR maneuver had a predictive value similar to that of a respiratory variation in pulse pressure greater than 12%. In contrast, an isolated basal LVETc value is not a reliable criterion for predicting response to fluid loading.

As underlined by recent recommendations, dynamic criteria are better predictors of volume responsiveness than static criteria [14]. In this way, PLR, which is a simple, dynamic and reversible maneuver, has been proposed by Boulain and colleagues [11] to predict fluid responsiveness in mechanically ventilated patients. These authors reported a strong correlation between SV variations measured by thermodilution during VE and those induced by PLR. These results were also positively correlated with respiratory variations in pulse pressure simultaneously measured by an arterial catheter. However, no threshold value of PLR-induced changes in pulse pressure was proposed to predict fluid responsiveness in this study. Moreover, pulse pressure does not depend only on SV; it may also vary with arterial compliance and with the site of measurement. We hypothesized that a PLR maneuver monitored by an ED could reliably and non-invasively predict fluid responsiveness. Indeed, the ability of ED to detect a response to fluid loading has been demonstrated in a recent study [12]. ED has also been used to optimize fluid loading during the perioperative period. In four studies, this practice showed a benefit on postoperative length of stay in hospital [15-18]. Our study confirmed the ability of a PLR maneuver monitored by ED to predict fluid responsiveness. Interestingly, this PLR maneuver induced ABF changes that were similar in magnitude to that observed after a VE of 250 ml. This agrees with a previous study that showed a 300 ml shift in blood volume towards the intrathoracic compartment [7]. Our study permits a better definition of a threshold value for predicting fluid responsiveness. A PLR-induced increase in ABF of more than 8% predicted fluid responsiveness with a PPV of 82% and an NPV of 91%.

We found that a ΔPP threshold value of 12% offers the best sensitivity:specificity ratio to predict fluid responsiveness (sensitivity 70%, specificity 92%). However, the PPV and NPV of ΔPP (87% and 78%, respectively, for a 12% ΔPP) were not as high as previously described: Michard and colleagues [4] found a ΔPP threshold value of 13% offering a PPV of 94% and a NPV 96% for the prediction of fluid responsiveness. Many factors could explain such a difference. In the responder group we found a median basal ΔPP of 15%, whereas a mean of 24% was reported in the latter study [4]. This suggests a lower preload dependence level in our population. Moreover, it has been suggested that the magnitude of SV respiratory variations could be affected by the magnitude of tidal volume used [19]. Recently, De Backer and colleagues [20] also showed ΔPP to be a reliable predictor of fluid responsiveness only when tidal volume is at least 8 ml/kg. Thus, in our patients, the preload dependence state might have been underestimated, given the relatively low median tidal volume used (7 ml/kg). In this way, it can be noticed that two responders with a very low ΔPP were ventilated with 6 ml/kg tidal volume.

For several authors, LVETc may constitute an index of left ventricular preload. Singer and colleagues [2,21] investigated LVETc as a measure of ventricular filling by placing an ED and a pulmonary artery catheter in either healthy volunteers or cardiac surgery patients. The authors observed a matched increase in pulmonary capillary wedge pressure and LVETc after fluid loading in all patients with hypovolemia. Similarly, all normovolemic patients had a concordant decrease in pulmonary capillary wedge pressure and LVETc when preload was decreased. In the same way, Madan and colleagues [3] conducted a study in 14 surgical critically ill patients and found a better correlation between thermodilution-measured cardiac output and LVETc (*r *= 0.52) than between thermodilution-measured cardiac output and pulmonary capillary wedge pressure (*r *= 0.2). More recently, Seoudi and colleagues [22] also suggested the superiority of LVETc on pulmonary capillary wedge pressure in assessing preload status, especially in patients ventilated with a high positive end-expiratory pressure. In coronary bypass surgery patients, DiCorte and colleagues [23] found a better correlation between LVETc and end-diastolic short-axis area as measured by transesophageal echocardiography (*r *= 0.49) than between pulmonary artery diastolic pressure and end-diastolic short-axis area (*r *= 0.10). Our findings are in accordance with those of Singer and colleagues.

In responder patients, LVETc increased during PLR and VE but did not change in non-responders. However, we found an isolated basal LVETc value to be a poor predictor of ABF response to fluid loading. In a recent study of critically ill patients with acute circulatory failure, Monnet and colleagues [5] showed that the respiratory variation in peak aortic velocity or ABF provides a better prediction of fluid responsiveness than LVETc, which was not a reliable indicator of fluid responsiveness in their population. Our findings are in accordance with these results. In our patients, whatever the threshold value, the predictive value of LVETc was also much lower than those of our dynamic criteria (ABF variations induced by PLR or ΔPP) to discriminate between responders and non-responders. This result can be explained in several ways. In patients in intensive care, acute circulatory failure is a complex hemodynamic condition that leads to frequent changes in preload, afterload and inotropic state. LVETc is a static criterion that is influenced not only by preload conditions but also by afterload level [21]. Moreover, acute circulatory failure often requires the introduction of vasopressive drugs that affect heart rate; this raises questions about the adjustment calculation of LVETc. Finally, our results show that an isolated value of LVETc is not a reliable index for predicting fluid responsiveness.

Our study does have some limitations. The results were obtained from only a small number of patients and the study is underpowered to permit a definitive conclusion. With regard to the power of our fluid challenge to discriminate between responders and non-responders, we infused only 500 ml of crystalloids; other authors have used larger amounts of fluids. In addition, considering that vasopressors were used in all these patients, several of them might have been classified as non-responders just because they did not receive sufficient fluids to affect their preload [24]. It is possible that conducting a larger volume expansion might have led us to identify more responders. However, our data are consistent with recently published findings in which the authors employed similar volumes of fluids [25]. These conclusions can also be applied only to mechanically ventilated patients receiving low tidal volumes. Furthermore, although ED offers a continuous ABF measurement, a repositioning of the probe is often necessary to maintain the best signal, especially during PLR. The use of a Trendelenburg position might be an easier method; this should be explored in future studies. Finally, we did not test intraobserver and interobserver variability in the ABF measurement. As suggested by Roeck and colleagues [12], this could alter the precision and reproducibility of the PLR maneuver in detecting fluid responsiveness.

## Conclusion

Our data support the non-invasive assessment of ABF variations provoked by a PLR manoeuver in the prediction of fluid responsiveness in mechanically ventilated patients with acute circulatory failure. The predictive value of this test is comparable to that of ΔPP. In contrast, an isolated static LVETc value furnished by ED devices is not a reliable criterion for predicting response to fluid loading.

## Key messages

• PLR is a simple reversible maneuver that mimics a rapid fluid loading.

• We found that a PLR-induced increase in ABF greater than 8% predicted the response to subsequent VE with a sensitivity of 90% and a specificity of 83%.

• An isolated basal LVETc value is not a reliable criterion for predicting response to fluid loading.

• Fluid responsiveness in mechanically ventilated patients with acute circulatory failure can be efficiently predicted by assessing the effects of PLR on ABF monitored by ED.

## Abbreviations

ABF = aortic blood flow; AUC = area under the receiver operating characteristic curve; ΔPP = respiratory pulse pressure variation; ED = esophageal Doppler; LVETc; left ventricular ejection time corrected for heart rate; NPV = negative predictive value; PLR = passive leg raising; PPV = positive predictive value; ROC = receiver operating characteristic; SV = stroke volume; VE = volume expansion.

## Competing interests

The authors declare that they have no competing interests.

## Authors' contributions

AL and AC designed the study. AL, FP, CG, AD, VM and GC realized the experiments. JDC, JPM and AC contributed to data collection. AL, FP and AC wrote the manuscript. All authors participated in its critical revision. AC had full access to all data in the study and had final responsibility for the decision to submit for publication. All authors read and approved the final manuscript.

## Figures and Tables

**Table 1:** Patient characteristics.

Characteristic	Patients (*n *= 22)
Age (years)	69 (56–73)
Male/female	13/9
Diagnosis	
Severe sepsis	1
Septic shock	13
Hemorragic shock	2
Post-resuscitation syndrome	4
Acute poisoning	2
Vasopressive agents	
Norepinephrine	10
Epinephrine	11
Norepinephrine (μg/kg per minute)	0.6 (0.3–0.7)
Epinephrine (μg/kg per minute)	0.4 (0.3–1)
Ventilator settings	
Tidal volume (ml/kg)	7 (6.7–8.5)
PEEP (cmH_2_O)	8 (5–10)

Results are shown as median (interquartile) or as number. PEEP, positive end-expiratory pressure.

**Table 2:** Hemodynamic parameters in VE responders.

Parameters	Base 1	PLR	Base 2	VE 250 ml	VE 500 ml
HR (beats/minute)	106 (104–114)	107 (103–113)	109 (104–112)	104^b ^(100–113)	105^c ^(101–113)
SAP (mmHg)	102 (87–129)	103 (91–133)	103 (89–127)	107 (102–133)	114^c ^(104–139)
MAP (mmHg)	75 (59–87)	76 (61–89)	74 (60–83)	81 (67–86)	82^c ^(69–89)
DAP (mmHg)	54 (46–67)	58 (47–68)	56 (46–67)	58 (49–68)	61 (49–66)
ABF (l/minute)	2.4 (1.8–4)	2.8^a ^(2–4.9)	2.6 (1.8–3.7)	2.9^b ^(2.6–5)	3.4^c ^(2.7–5.1)
LVETc (ms)	232 (209–272)	248^a ^(232–282)	230 (209–266)	250^b ^(233–296)	259^c ^(239–295)
ΔPP (percentage)	15 (12–17)	10^a ^(8–13)	13 (9–16)	-	8^c ^(4–11)

Results are shown as median (interquartile). PLR, passive leg raising; VE, volume expansion; HR, heart rate; SAP, systolic arterial pressure; MAP, mean arterial pressure; DAP, diastolic arterial pressure; ABF, aortic blood flow; LVETc, left ventricular ejection time corrected for heart rate; ΔPP, respiratory variation of pulse pressure (see the text). ^a^*p *< 0.05 PLR versus Base 1, ^b^*p *< 0.05, VE 250 ml versus Base 2, ^c^*p *< 0.05, VE 500 ml versus Base 2.

**Table 3:** Hemodynamic parameters in VE non-responders.

Parameters	Base 1	PLR	Base 2	VE 250 ml	VE 500 ml
HR (beats/minute)	97 (79–121)	96 (80–120)	104 (78–22)	92 (79–121)	91 (79–121)
SAP (mmHg)	115 (108–135)	118 (110–138)	114 (105–130)	115 (105–126)	119 (109–128)
MAP (mmHg)	68 (62–91)	71 (67–95)	66 (63–86)	69 (62–80)	72 (63–82)
DAP (mmHg)	50 (41–74)	52 (45–72)	48 (41–67)	53 (44–61)	53 (45–63)
ABF (l/minute)	4.2 (3.3–4.9)	4.2 (3.3–5.3)	4.1 (3.4–5)	3.9 (3.4–5)	4.1 (3.4–5.1)
LVETc (ms)	259 (242–289)	264 (240–292)	270 (242–295)	265 (244–286)	273 (241–293)
ΔPP (percentage)	9 (5–10)	7^a ^(5–7)	8 (5–9)	-	5 (4–9)

Results are shown as median (interquartile). PLR, passive leg raising; VE, volume expansion; HR, heart rate; SAP, systolic arterial pressure; MAP, mean arterial pressure; DAP, diastolic arterial pressure; ABF, aortic blood flow; LVETc, left ventricular ejection time corrected for heart rate; ΔPP, respiratory variation of pulse pressure (see the text). ^a^*p *< 0.05, PLR versus Base 1.
